# Taxonomic and functional shifts in the rumen microbiome of buffalo calves under long-term strategic supplementation of phyto-feed additives

**DOI:** 10.3389/fvets.2025.1647762

**Published:** 2025-11-05

**Authors:** Pramod Kumar Soni, Anju Kala, Payal Agarwal, Rampratim Deka, Habibur Rahman, Kennady Vijayalakshmy, Lal Chandra Chaudhary

**Affiliations:** ^1^Department of Animal Nutrition, College of Veterinary and Animal Science University, Rani Lakshmi Bai Central Agricultural University, Jhansi, India; ^2^Animal Nutrition Division, ICAR-Indian Veterinary Research Institute, Izatnagar, India; ^3^ILRI, South East Asia Office, New Delhi, India

**Keywords:** phyto-feed additives, next-generation sequencing (NGS), functional metagenomics, microbial taxonomic shift, buffalo calves

## Abstract

**Introduction:**

The present study aimed to understand the shift in the rumen microbiome of buffaloes fed diets with and without phyto-additives. The rationale was based on the hypothesis that plant-based additives can modulate the microbial population in the rumen, potentially reducing methane production and enhancing fiber degradation. Given the possibility that prolonged use of the same additives may lead to microbial adaptation and diminished efficacy, the study also investigated the effects of periodically switching additives.

**Methods:**

Three male buffalo calves were fed a control diet, while another three received additive-supplemented diets. Two additive formulations were used: FAI (a blend of garlic *Allium sativum*, ajwain *Trachyspermum ammi*, harad *Terminalia chebula*, and soapnut *Sapindus mukorossi*) and FAII (ajwain oil). The additives were alternated every 15 days to prevent microbial adaptation. After 21 days of feeding, rumen liquor samples were collected 2 hours post-feeding for metagenomic analysis. The study included both *in vivo* and *in vitro* assessments of rumen fermentation.

**Results:**

Metagenomic analysis revealed that dominant bacterial phyla included *Prevotella, Bacteroides, Succiniclasticum, Fibrobacter, Clostridium, Alistipes, Ruminococcus*, and *Butyrivibrio*, with over 50 bacterial species consistently present across all animals. The main archaeal phylum was *Euryarchaeota* (>85%), along with notable presence of *Candidatus_Bathyarchaeota* and *Thaumarchaeota*. At the genus level, *Methanomicrobium* and *Methanobrevibacter* each accounted for approximately 30% of the archaeal community, followed by *Methanosphaera*, *Methanosarcina*, and *Methanomassiliicoccus*. While total abundances of Archaea and Bacteroidota were not significantly different among groups, specific taxa within these phyla showed marked changes.

**Discussion:**

The inclusion of phyto-additives in the buffalo diet influenced the rumen microbiome composition by reducing methanogen populations, particularly *Methanobrevibacter*, and enhancing fiber-degrading microbial communities. These microbial shifts were associated with improved fiber utilization and decreased methane emissions. Rotating the additives every 15 days appeared to sustain their efficacy over time, potentially by preventing microbial adaptation. This approach may offer a sustainable strategy to optimize rumen function and reduce enteric methane emissions in ruminants.

## Introduction

Metagenomics is the analysis of the entire genetic material of microbiota in a living environment and is an important way to understand the taxonomic and functional characterization of intestinal microbiota. Animal gut microbiota, often termed the second genome, exhibit a strong association with host genetics. The bovine gastrointestinal system harbors a rich and diverse microbial community, where microbes from the rumen and gut play a critical role in host metabolism and overall health. These microbes facilitate energy production by fermenting undigested feed, and even slight alterations in their composition can profoundly influence nutrient absorption and livestock productivity. Numerous studies have reported that microbiota present in the gastrointestinal tract is closely related to cattle lactation, fat deposition, feed efficiency, and disease (1, 2). Although metagenomics has been used in various animals, such as humans, pigs, and mice, few studies using it to investigate cattle with different RFI phenotypes and correlated microbiota abundance with host gene expression have been reported.

The unique fermentation capability of the rumen is bestowed on microbes residing in their gut. These microbes produce an array of enzymes that utilize the forage and feed and, in turn, produce metabolites such as volatile fatty acids (VFAs), ammonia, lactic acid, CO_2_, methane, and hydrogen. These metabolites are the precursors for animals’ maintenance and their products. In recent years, plant secondary metabolites (PSMs) have long been claimed to favorably modulate rumen microbiome and its fermentation. Samal et al. (3) reported reduced methane and better feed efficiency among buffaloes fed with garlic, ajwain, harad, and soapnut. Wadhwa and Bakshi (4) and Chaturvedi et al. (5) found similar benefits with ajwain oil and herbal blends. A 2025 goat study showed a 22–33% lower methane with ajwain oil and bahera (6). However, the effect of these PSMs has been highly variable due to differences in the active principles, making it difficult to make concrete (7). In such a scenario, it becomes imperative to assess the PSMs under *in vivo* conditions to see how the PSM changes the rumen microbiome, fermentation pattern, and what degree of interdependence or correlations exist between these factors. In the present study, a blend of phyto feed additives using *Allium sativum* (garlic) bulb*, Trachyspermum ammi* (ajwain) seeds, essential oil*, Terminalia chebula* (harad) pulp, and *Sapindus mukorossi* (soapnut) nuts was used to study the above-mentioned objectives. *Allium sativum* is known for its organosulfur compounds such as allicin, diallyl sulfide, and ajoene, which possess antimicrobial, antioxidant, and immunomodulatory effects (8, 9). *Trachyspermum ammi* seeds and essential oil are rich in thymol, *γ*-terpinene, and p-cymene, offering strong antimicrobial, antifungal, and digestive properties (10, 11). *Terminalia chebula* contains tannins, gallic acid, chebulagic acid, and ellagic acid, which exhibit potent antioxidant, antimicrobial, and gut-toning effects (12, 13). *Sapindus mukorossi* is primarily composed of saponins and triterpenoids, known for their surfactant, anti-protozoal, and microbial population-modulating properties (14, 15). Together, these phytogenic additives offer a wide range of secondary metabolites that can potentially alter the microbial ecosystem of the rumen, suppress methanogenesis, enhance fermentation efficiency, and improve nutrient utilization.

## Materials and methods

### Experimental animals

Six male buffalo calves, 12–15 months of age with an average body weight of 165 ± 4 kg, were divided into two groups based on body weight in a completely randomized design. Animals were housed in the Animal Nutrition shed of the Indian Veterinary Research Institute, Izatnagar. Prior to the initiation of the experiment, animals were treated against ecto- and endo-parasites according to the standard protocol. The feeding trial was continued for 6 months.

### Feeding management and treatment

Animals were kept individually in a well-ventilated shed with a cement floor and were fed a concentrate mixture and wheat straw in a 50:50 ratio, targeting a growth rate of 500 g/d as per ICAR (2013). The concentrate mixture of 20% protein comprised 35% maize, 24% soybean meal, 38% wheat bran, 2% mineral mixture, and 1% salt on a DM basis. The wheat straw was offered after the concentrate mixture was consumed. To meet the vitamin requirement, 5 kg of chopped green maize per animal was provided once a week. Fresh and clean drinking water was made accessible *ad libitum* twice a day, at 9 AM and 5 PM.

The additives used in the present study were FAI, a blend of garlic (*Allium sativum*), ajwain (*Trachyspermum ammi*), harad (*Terminalia chebula*), and soapnut (*Sapindus mukorossi*) in equal proportion at the rate of 1% of DMI and FAII, ajwain oil, at a rate of 1 mL/kg DMI alternatively every 15 days for 6 months. The FAs were mixed well with the concentrate mixture. The two treatments were Con- Control and Treat-Treatment, which were fed alternatively every 15 days for 6 months. The additive PFAI was a powder of *Allium sativum* bulb (garlic), *Trachyspermum ammi* seeds (ajwain), *Terminalia chebula* pulp (harad), and *Sapindus mukorossi* nut (soapnut), mixed in equal proportion, and PFAII was *Trachyspermum ammi* essential oil. The four plant parts used in PFAI were dried, powdered, and then mixed in equal proportions. The PFAs were mixed well with the concentrate mixture daily for the entire experimental period and ensured the complete consumption of conc. mix. Chopped wheat straw was offered after the concentrate mixture was completely consumed.

### Sampling of rumen liquor

The rumen liquor was collected from three buffaloes of each group by using a stomach tube at the end of the feeding trial to assess the diversity of the rumen microbiome and its related functions. The rumen liquor was collected 2 h after feeding and then mixed with an equal volume of RNA later and then stored at - 80°C till further use. After that, next-generation sequencing for all the samples was performed on the Illumina Platform with a chemistry of 2 × 150 base pair (bp). The master denovo assembly to prepare contigs was carried out for all six samples using CLC Genomics Workbench v6.1, and FASTA sequences were generated. The statistical components of the assemblies were determined using custom Perl scripts developed in-house.

### Gene prediction

The master assembled transcripts were subjected to coding gene prediction using Prodigal (v2.6.3), considering the metagenome gene prediction method. To determine the genes expressed in each sample, reads of each sample were mapped to these predicted genes using bwa v0.7.12. The genes having reads mapped were considered expressed for that particular sample. The identified genes for each sample were then subjected to further taxonomic and functional analysis.

### Taxonomic annotation of CDS

Kaiju is an efficient and standalone metagenome classifier that uses the Burrows–Wheeler transform algorithm to detect the highest exact protein-level matches. It classifies individual metagenomic reads by referencing a database of annotated protein-coding genes from various microbial genomes, which are primarily sourced from NCBI RefSeq. The final gene sequences obtained from prodigal were taken as input in Standalone Kaiju for assessing the taxa of the rumen microbial sequences under study with Database-Nr, Run mode-Greedy, Minimum match score-75, and Allowed mismatches-5. The output file obtained from KAIJU, having taxonomy assigned information, was converted to OTU and taxa using R. Eventually, these data files in control and treatment were converted into a phyloseq object to derive alpha diversity using “estimate_richness” of the phyloseq package. Alpha diversity was obtained in terms of Chao1, Shannon, Simpson, and Inv Simpson, and was expressed in the form of a boxplot using the “plot_richness” function. Data filtration and normalization were performed, and the OTUs were divided into the Bacteria and Archaea domains. Other taxonomy analyses for the phylum, genus, and species were carried out using MicrobiomeAnalyst, using the OTU table, taxa table, and metadata files. Feature filtration was performed for bacteria as well as archaea using a low-count filter with a minimum count of 2 and a prevalence of 10% of the samples. A low variance filter was applied to remove features having an inter-quantile range (IQR) of 5%. Core microbiome analysis was carried out using MicrobiomeAnalyst using the following criteria: sample prevalence (%): 20% relative abundance (%): 0.01. The core microbiome was identified at the genus level for the bacterial domain.

### Network analysis for microbial genus and functional parameters

Correlation Network was constructed at the phylum and class level for the bacterial domain using the Spearman’s rank correlation algorithm at a *p*-value threshold of 0.001 and a correlation threshold of 0.5. For archaea, a correlation network was constructed at the phylum and class levels for the archaeal domain using the Spearman’s rank correlation algorithm at a default *p*-value threshold of 0.05 and a correlation threshold of 0.3. Here, each node represents a taxon with its color based on the mean abundance and its size based on the number of connections to that taxon. Two taxa are connected by an edge if the correlation between the two taxa meets the *p*-value and correlation threshold. The edge size also reflects the magnitude of the correlation. Green color represents the control group, whereas orange color represents the treatment group.

### CAZyme analysis

The CAZyme database was downloaded from dbCAN2. The protein sequences obtained from the prodigal were searched for similarity using BlastP against CAZyDB with an E-value of 1^e-06^. To estimate CAZyme profiles, reads were mapped to CDS having CAZy information using bwa v0.7.12 and quantified using Samtools v1.4.1. Thus, the abundance of all CAZymes was calculated. A matrix having 604 CAZyme families with read count for all six samples was provided as the shotgun data profiling (SDP) option of MicrobiomeAnalyst to calculate PCA, LEfSe, and differential analysis. The features for CAZymes were filtered at default parameters, i.e., a low count filter of minimum count 4, i.e., a low count filter of minimum count 4, and prevalence in 20% samples was applied, i.e., for any feature to be retained, at least 20% of its values should contain at least 4 counts. By default, a low variance filter was applied to remove features having an inter-quantile range (IQR) of 10%. Some features were removed based on low abundance and prevalence. Finally, these filtered features were normalized using the default option of cumulative sum scaling (CSS) and used for analysis.

### Diversity at the phylum, order, family, genus, and species levels for bacteria

#### Bacterial domain

Due to the presence of a large number of classifications present for the bacterial domain, the groupwise top 50 phylum, class, order, family, genus, and species were plotted for ease of representation. Furthermore, linear discriminant analysis (LDA) effect size (LEfSe) was estimated at phylum and genus levels for the bacterial domain using the LEfSe option of MicrobiomeAnalyst with a *p*-value of 0.05 and log LDA score of 2. The top 15 significant differential features at the phylum and genus levels were plotted.

### Diversity at the phylum, order, family, genus, and species levels for archaea

#### Archaeal domain

As very limited OTUs were representing the archaeal domain, group-wise all phylum, class, order, family, genus represented by the archaeal domain were plotted, except for species. At the species level, the top 100 most abundant were plotted for ease of presentation. Linear discriminant analysis (LDA) effect size (LEfSe) was estimated at the phylum, genus, and species levels for the archaeal domain using the LEfSe option of MicrobiomeAnalyst with a *p*-value of 0.05 and a log LDA score of 2. Significant differential features at the phylum, genus, and species levels plotted were 12, 16, and 15, respectively.

#### Principal coordinate analysis for bacterial genus and archaeal species

The distance method used was the Bray-Curtis index, the taxonomic level was genus for bacteria and species for archaea, and the statistical method used was permutation MANOVA (PERMANOVA).

#### Functional annotation

To study the functional relationship to the observed rumen microbiome and to assess the functional capacities of microbial communities, functional annotation was conducted using COGNIZER (v0.9b) at default parameters, taking the predicted genes for each sample as input. Cognizer is an all-in-one, standalone framework designed to simultaneously deliver COG, KEGG, Pfam, GO, and FIG fam annotations for individual sequences within metagenomic datasets. The results obtained from Cognizer were further analyzed using MicrobiomeAnalyst.

### KEGG pathway analysis

For metabolic pathway analysis, reads were mapped to CDS having KEGG information using bwa v0.7.12 and quantified using Samtools v1.4.1. Thus, the abundance of all pathways was calculated. A matrix having 3,866 KO IDs with read count for all six samples was provided as the shotgun data profiling (SDP) option of MicrobiomeAnalyst to calculate PCA, LEfSe, and differential analysis. The features were filtered using default parameters, including a low count filter (minimum count of 4 and presence in at least 20% of the samples). Additionally, a low variance filter was applied to remove features with an inter-quantile range (IQR) below 10%. Finally, these filtered features were normalized using the default option of cumulative sum scaling (CSS) and used for analysis. Functional diversity profiling was carried out for KEGG KO IDs. The category abundance was calculated based on total hits and grouped by control and treatment. Functional diversity profiling was carried out using three functional categories, namely KEGG metabolism, KEGG pathways, and COG functional categories.

### Linear discriminant analysis (LDA) effect size (LEfSe)

LEfSe analysis was carried out at a *p*-value cutoff of 0.05 and a log LDA score of 2.0. A total of 28 significant features were found and plotted in the form of a barplot. It contained a total of 15,666 bacterial OTUs (OTUs with count > = 2 are 13,811) and 433 archaeal OTUs (OTUs with count > = 2 are 369).

### Feature filtration

After filtration, the following were the stats:

(A) For bacteria a total of 3,977 low-abundance features were removed based on prevalence. A total of 492 features exhibiting low variance were discarded based on the interquartile range (IQR). The number of features remaining after the data filtration step is 9,342. Finally, these filtered features were normalized using default, total sum scaling (TSS), and used for analysis.
(B) For archaea a total of 184 low-abundance features were removed based on prevalence. A total of 10 low-variance features were removed based on IQR. The number of features remaining after the data filtration step is 175. Finally, these filtered features were normalized using default, total sum scaling (TSS), and used for analysis.

### Statistical analysis

Alpha diversity was derived using “estimate_richness” of the phyloseq package. Taxonomy analysis was carried out using MicrobiomeAnalyst using OTU table, taxa table, and metadata file.

## Results and discussion

Metagenomic analysis of a highly complex rumen microbiome provides a holistic approach to explore both cultivable and non-cultivable microbes. This experiment aimed at studying the changes in the microbial community structure of rumen microbiota patterns through the supplementation of additives in buffaloes.

### Diversity at the phylum, order, family, genus, and species levels for bacteria

**Figure 1:**
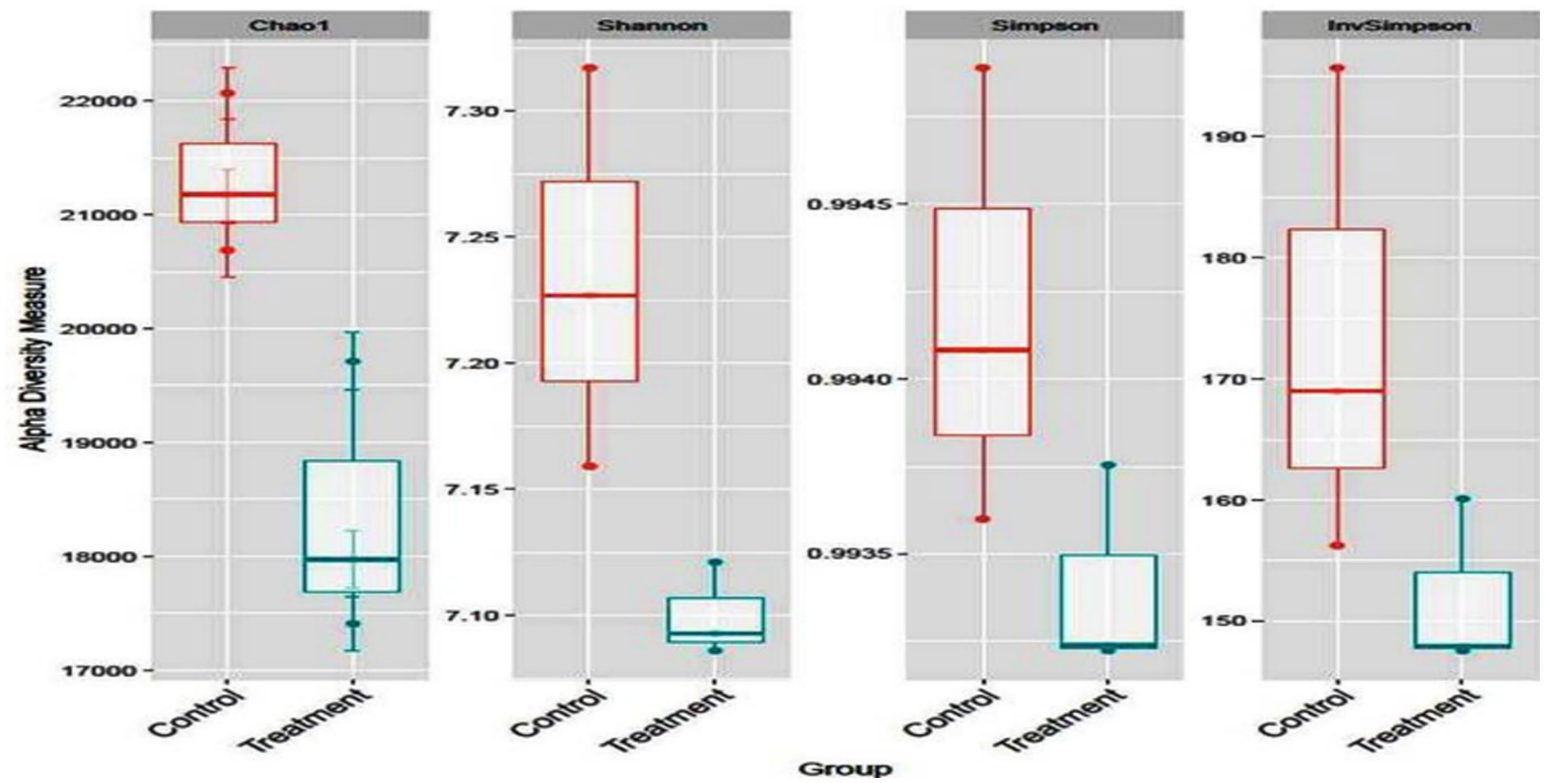
Alpha diversity of the buffalo rumen microbiome fed additive.

Alpha diversity was obtained in terms of Chao1, Shannon, Simpson, and Inv Simpson. It was similar across all the groups (*p* = 0.093) showing a trend where higher diversity was obtained for the treatment group than control (Figure 1). Many metagenomic studies on the rumen microbiome reported similarity of abundance of alpha and beta diversity of rumen microbiome and predominance of Bacteroidetes and Firmicutes (at the phylum level) and *Prevotella* (at genus level) in buffaloes fed on various proportions of concentrate and roughage (28) and TDN (16) and in nitrate supplemented steers at 0, 1, and 2% (29).

### Bacterial domain

**Figure 2:**
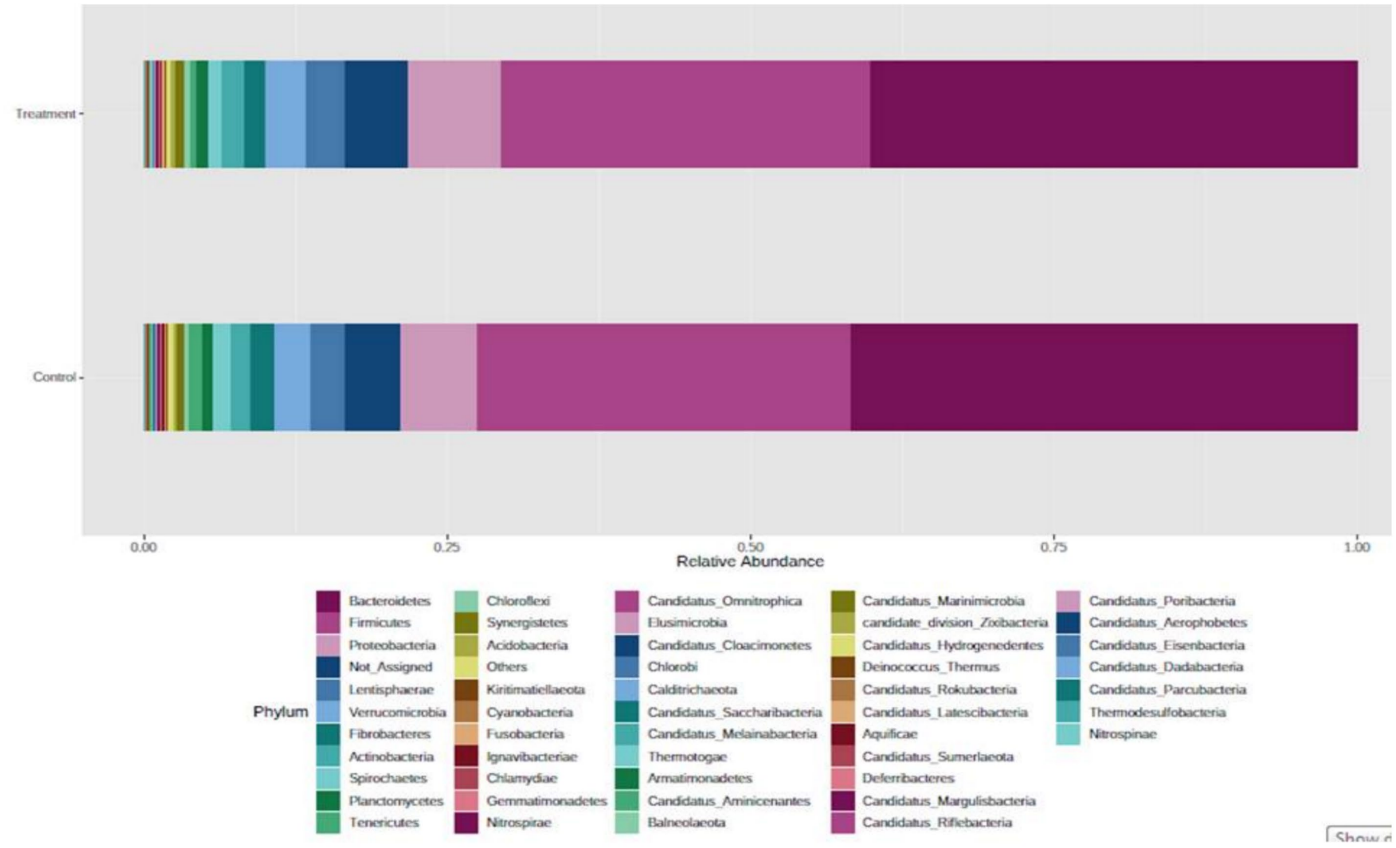
Abundance of bacteria at the phylum level in the buffalo rumen microbiome fed additive.

In the bacterial domain, at the phylum level, *Bacteroidetes* was the most abundant phylum, followed by *Firmicutes*, *Proteobacteria*, *Lentisphaerae*, *Verrucomicrobia*, *Actinobacteria,* and *Fibrobacteres* (Figure 2). At the class level, *Bacteroidia* was most abundant, followed by *Clostridia*, *Gammaproteobacteria, Negativicutes*, *Bacilli*, *Flavobacteria*, and *Fibrobacteria*. A good proportion of bacteria were placed in the not assigned category, indicating that the rumen microbes remain unknown at large. Similar to our observations, Nardi et al. (17) reported that bacterial phyla, such as *Bacteroidetes, Firmicutes, Tenericutes*, and *Euryarchaeota,* were abundant (> 1%), and *Fibrobacteres* were less abundant (0.1–1%) in the rumen of control, polyphenol, and organic acid supplemented heifers. The highest abundance of *Bacteroidetes* and Firmicutes has been reported by several researchers (16, 18–20).

When mined at the family level, the most abundant chunk of reads was assigned to the ‘not assigned’ group, followed by families *Prevotellaceae*, *Lachnospiraceae*, *Bacteroidaceae*, *Ruminococcaceae,* and *Clostridiaceae*. These other top 50 family members are presented in Figure 2. At the genus level among the most abundant 50 genera, *Prevotella* was the major abundant genus after the ‘*not assigned’* group, followed by *Bacteroides, Succiniclasticum, Alistipes, Fibrobacter, Clostridium, Oscillibacter, Acinetobacter, Ruminococcus, Butyrivibrio,* and *Eubacterium. Fibrobacter* and *Ruminococcus* have long been thought to be the major microbes for fiber degradation in the rumen, but their lower abundance (%) hints that there are microbes in the rumen other than these well-established fibrolytic bacteria, which play a crucial role in fiber utilization. *Ruminococcus* breaks down hemicellulose, pectin, and cellulose in the plant cell wall by producing a variety of cellulolytic and hemicellulolytic enzymes (21). Similarly, Stevenson and Weimer (22) found that key fibrolytic bacteria, such as *R. flavefaciens, R. albus, and F. succinogenes,* accounted for only approximately 2% or less of the ruminal bacterial 16S rRNA. In the present study, *Bacteroides* was the second most abundant bacteria in the rumen, followed by *Prevotella*. Naas et al. (23) have reported that *Bacteroides* has cellulolytic activity and the presence of polysaccharide-utilizing loci (PUL), a system of lignocellulosic feed utilization other than extracellular and cell-bound cellulosomes in their genome. This finding strengthens the hypothesis of the presence of an alternate mechanism of fibre utilization (used by other rumen microbes) in the rumen other than that of *Fibrobacter* and *Ruminococcus* species.

Furthermore, the LDA and LEfSe were estimated at the phylum and genus levels for the bacterial domain, with a *p*-value of 0.05 and a log LDA score of 2, identified 18 significant phyla and 274 significant species that varied between the two groups. The top 15 significant differential features included *Bacteroidetes, Tenericutes, Spirochaetes,* and *Thermotogae* in control, whereas in treatment, *Proteobacteria, Lentisphaerae, Actinobacteria, Kiritimatiellaeota,* and *Chlamydiae* were found to differ in the two groups. At the genus level, *Succiniclasticum, Clostridium, Coprobacillus, Butyrivibrio, Paludibacter, Parabacteroides, Anaeroplasma* in the control, and *Oscillibacter, Acinetobacter, Escherichia, Enterococcus, Pseudoflavonifractor, Kangiella,* and *Flavonifractor* in the treatment group were different. In the rumen, a higher intake of dietary concentrate caused an increase in the abundance of *Proteobacteria*, while the levels of *Bacteroidetes* and *Firmicutes* were reduced (24).

The core microbiome assessment of the two groups was done individually and in combination to have an idea of the core group of rumen bacteria that were essentially present in all experimental animals. It was observed that in control, the core microbiome consisted of genera such as *Prevotella, Bacteroides, Succiniclasticum, Fibrobacter, Clostridium, Alistipes, Ruminococcus,* and *Butyrivibrio*. During treatment, the core microbiome remained similar to the control with some exceptions. In treatment, *Butyrivibrio* was not part of the treatment core microbiome, whereas *Oscillibacter* and *Actinobacteria* were additionally found in the core microbiome of treatment but not control. Furthermore, the abundance of *Fibrobacter* and *Ruminococcus* was higher in the control group than in the treatment group. However, the largest proportion of the core microbiome remained ‘*unassigned*’, showing that the core microbiome in ruminants comprises many species that remain unknown in large numbers. Hence, unknown microbes and the metabolic pathways they adopt may be the reason for not finding any change in the feed digestibility of animals despite a change in the relative number of important known fibre degraders in the treatment group compared to the control.

### Archaeal domain

**Figure 3:**
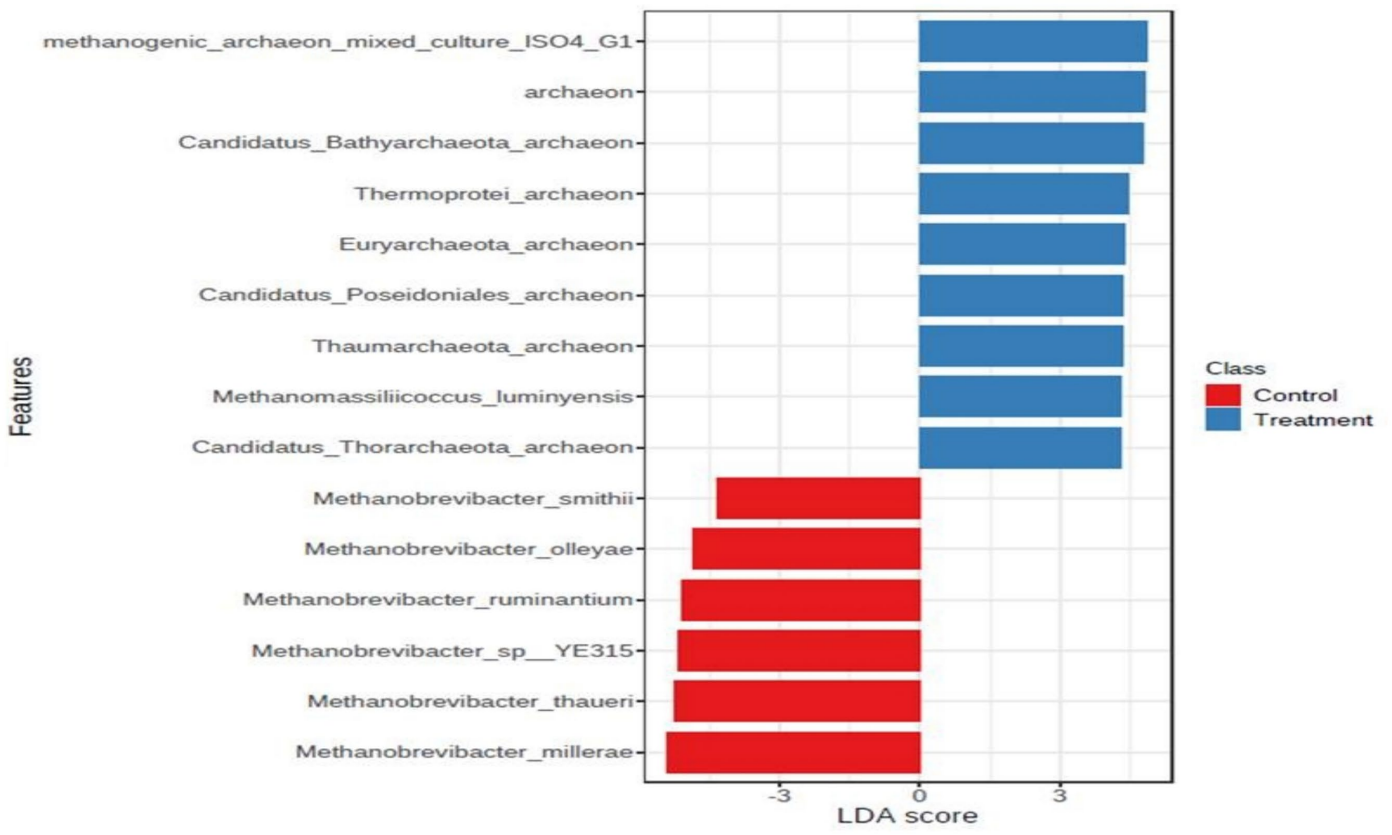
LDA score at the species level.

In archaea, at the phylum level, *Euryarchaeota* was the most abundant phylum, constituting more than 85% of the abundance. The other major phyla included *Candidatus_Bathyarchaeota, Crenarchaeota, Candidatus_Lokiarchaeota, Candidatus_Woesearchaeota,* and *Thaumarchaeota*. In the archaeal phylum, a very major share was possessed by the ‘*Not Assigned’* group. At the genus level, *Methanomicrobium* and *Methanobrevibacter* were the most abundant genera, to a tune of 30% each followed by *Methanosphaera, Methanosarcina,* the genus level, Methanomicrobium and Methanobrevibacter were the most abundant genera, to the tune of 30% each, followed by Methanosphaera, Methanosarcina, and *Methanomassiliicoccus*, to name a few. At the species level, *Methanomicrobium_mobile* was the most abundant species with an abundance of 30%. The other major species were methanogenic_archaeon_mixed_culture_ISO4_G1, *Methanomassiliicoccales archaeon* Mx 02, *Methanobrevibactermillerae*, *Methanobrevibacter_sp__YE315*, *Methanobrevibacter_thaueri*, *methanogenic_archaeon_ISO4_H5*, *Methanomassiliicoccales_archaeon*_Mx_06, *Methanobrevibacter_ruminantium*, *Methanobrevibacter_olleyae*, Methanosphaera_sp__BMS, *Methanobrevibacter_oralis*, *Methanosarcina_acetivorans*, and *Methanobrevibacter_smithii*. It was noticeable that the composition of archaea within the archaeal niche was different for control and treatment. At the phylum level, *Euryarchaeota* was more abundant in control than the phylum level, Euryarchaea were more abundant in the control than in the treatment. The Euryarchaeota phylum contains most of the methanogenic archaea, and its decrease in control indicates that the additive affected some selective species of the archaeal community. At the genus level, the abundance of *Methanobrevibacter* was lower in the treatment group than in the control. Interestingly, it appeared that the niche emptied by *Methanobrevibacter* was occupied by other archaea such as *Methanomicrobium* and some *unassigned* sequences, as their number was higher in the treatment group than control. Methanosphaera was also higher in treatment group than the control. Methanosphaera is known to utilize hydrogen without producing methane. So, it is evident that the supplementation of the additive restructured the archaeal abundance within the niche and decreased the abundance of microbes involved in methane production and favored microbes, which decreased methane emission. At the species level also, the species belonging to *Methanobrevibacter*, such as *Methanobrevibacter millerae*, *Methanobrevibacter_sp__YE315*, and *Methanobrevibacter_thaueri* were decreased in the treatment group compared to the control, clearly shown in the LDA score (Figure 3).

**Figure 4:**
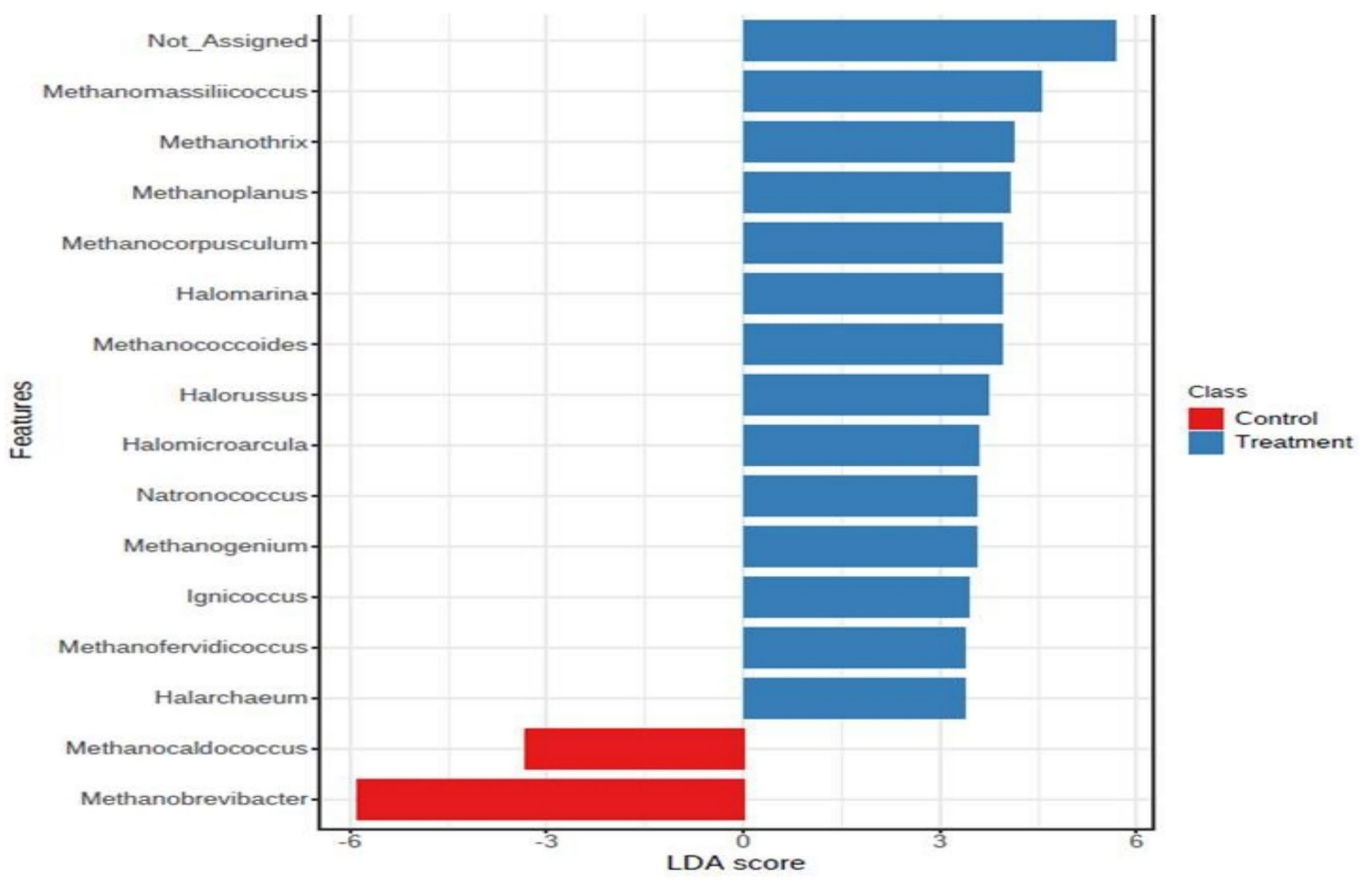
LDA score of control and treatment group.

The core microbiome of archaea at species level clearly showed that the core microbiome was majorly contributed by *Methanomicrobium_mobile, Methanobrevibacter millerae*, *Methanobrevibacter_sp__YE315*, *Methanobrevibacter_thaueri,* Methanobrevibacter_ruminantium, and *Methanobrevibacter_olleyae*, whereas the treatment core microbiome consisted of *Methanomicrobium_mobile*, *methanogenic_archaeon_mixed_culture_ISO4_G1, Methanomassiliicoccales_archaeon_Mx_02, Methanobrevibacter_millerae, Methanobrevibacter_sp__YE315, methanogenic_archaeon_ISO4_H5, Candidatus_Bathyarchaeota_archaeon, Methanobrevibacter_thaueri,* and *methanogenic_archaeon_ISO4_H5*, archaeon (Figure 4).

### KEGG pathway analysis

**Figure 5:**
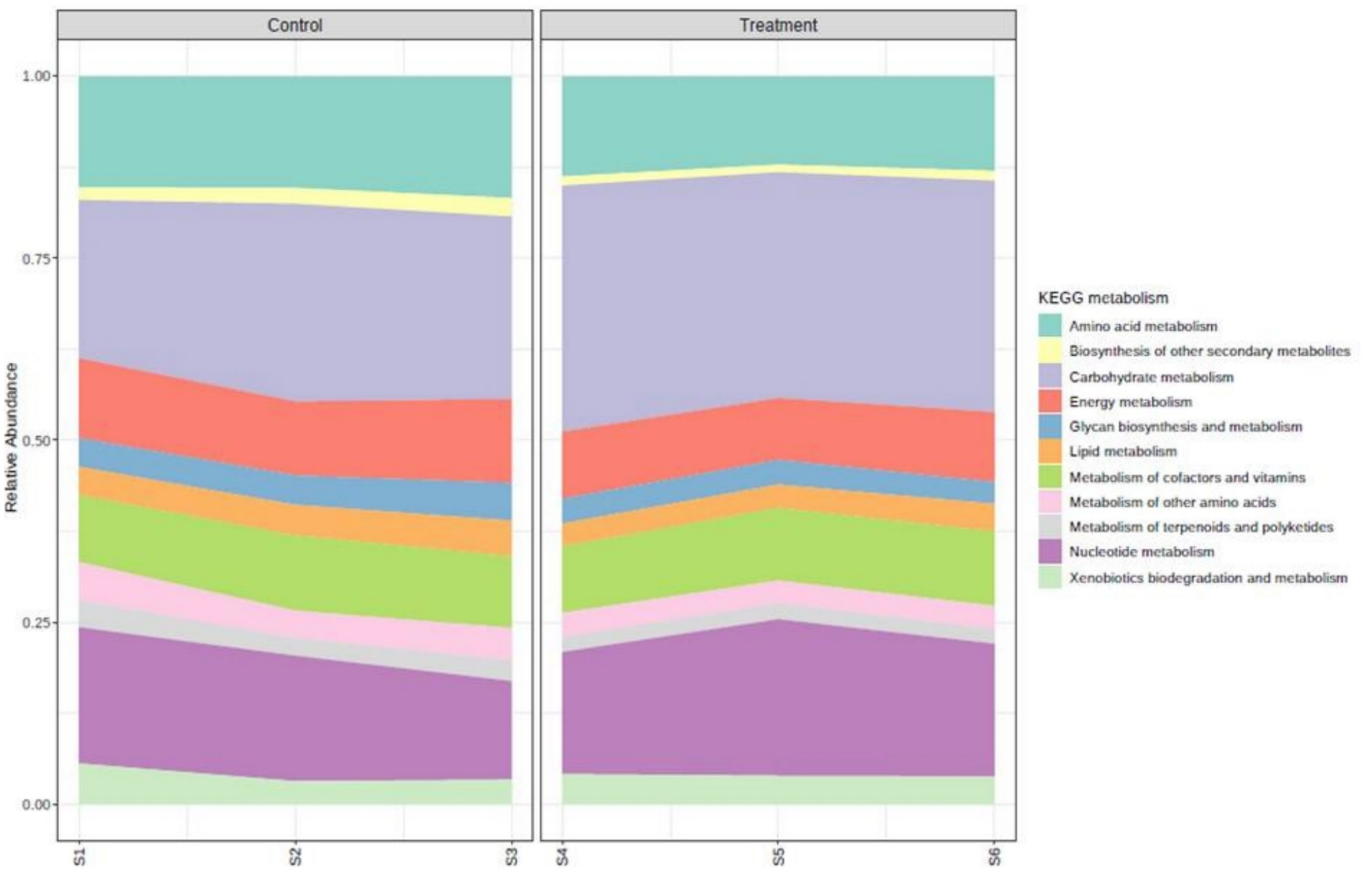
Major KEGG metabolism pathways for control and treatment group.

A total of 290 low-abundance features were removed based on prevalence. A total of 349 low-variance features were removed based on IQR. The number of features remaining after the data filtration step is 3,134. The KEGG pathway analysis revealed that a total of 133 pathways were observed to be expressed in the experimental animals without any effect of treatment (Figure 5). The major KEGG metabolism pathways that were highly expressed in order of abundance in both experimental groups were amino acid metabolism, biosynthesis of other secondary metabolites, carbohydrate metabolism, energy metabolism, glycan biosynthesis and metabolism, lipid metabolism, metabolism of cofactors and vitamins, metabolism of other amino acids, metabolism of terpenoids and polyketides, nucleotide metabolism, and xenobiotics biodegradation and metabolism. The expression of these pathways did not vary between the two groups.

LEfSe analysis was carried (*p*-value cutoff 0.05 and log LDA score of 2.0). A total of 28 significant features for KEGG metabolism pathways were found to be different in the treatment group than the control (Figure 5).

### CAZyme analysis

**Figure 6:**
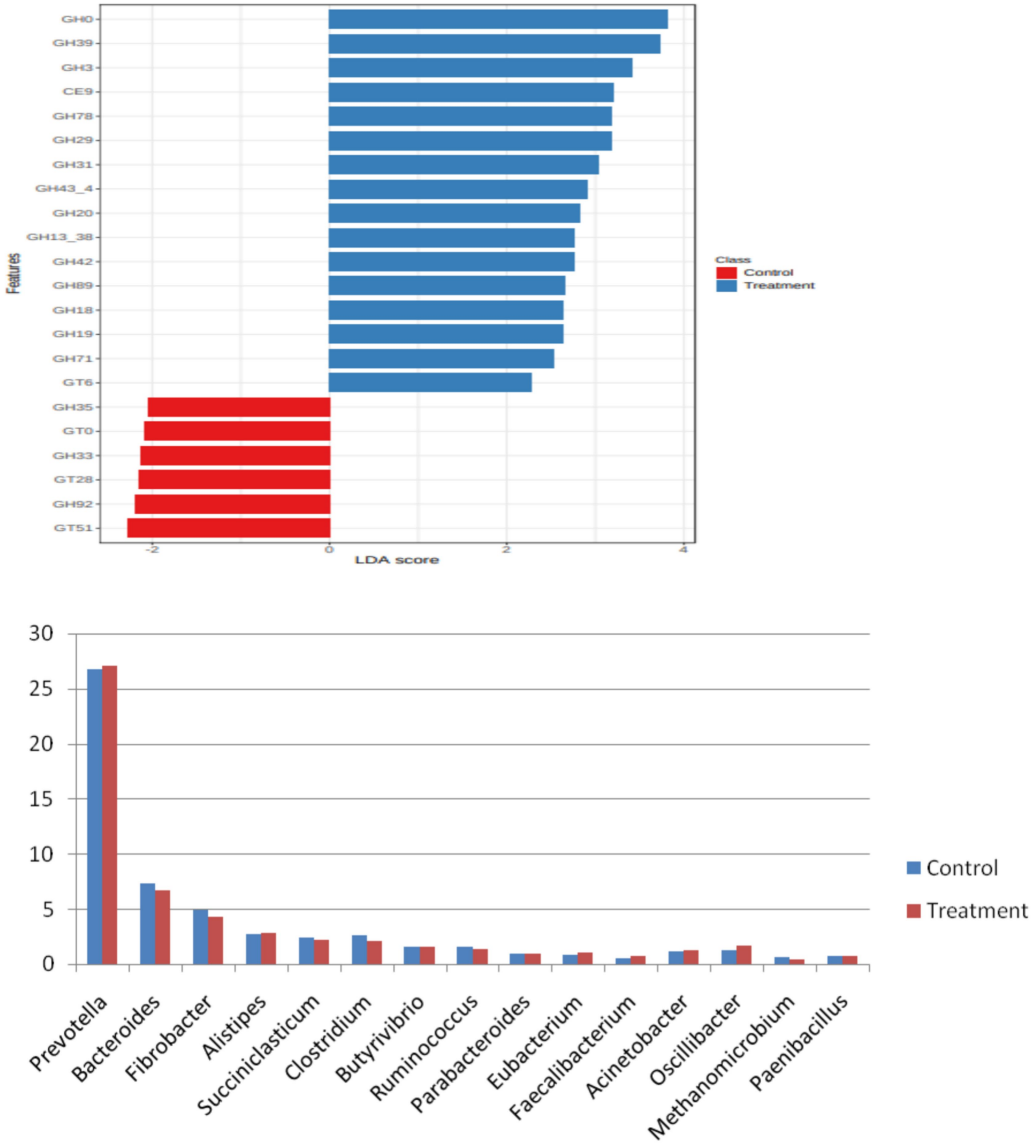
Genus CAZymes.

**Figure 7:**
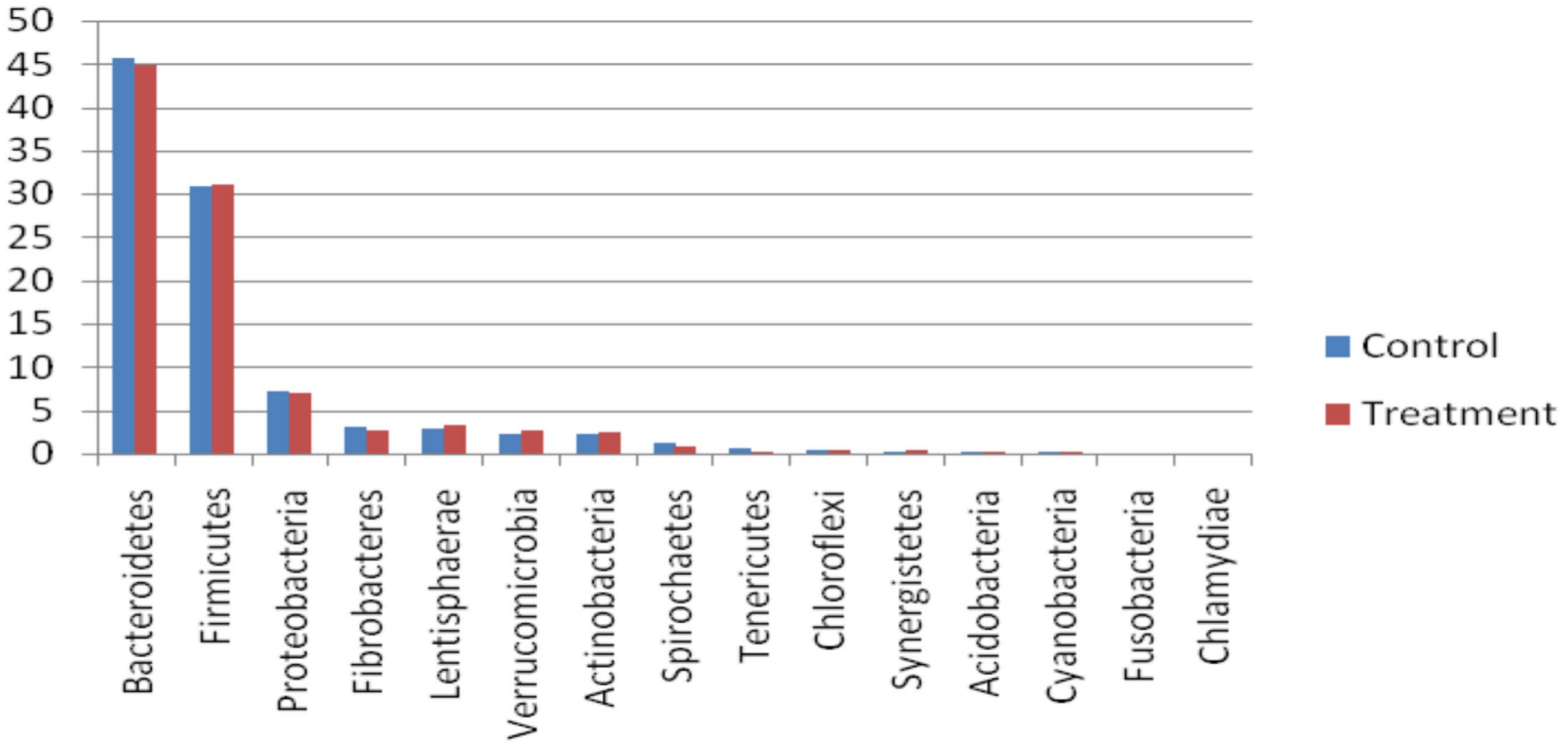
Phylum CAZymes.

Rumen fluid harbors complex of enzymes which work in synergistic manner to bring about fiber utilization and these enzymes are contributed by rumen microbes. The CAZyme class majorly includes glycoside hydrolases (GHs), which hydrolyze glycosidic bonds of carbohydrates, cellulose binding modules (CBMs), involved in enzyme substrate binding, carbohydrate esterases (CEs), which break the ester bonds between lignin and carbohydrates in the rumen. LEfSe analysis was carried out at a *p*-value cutoff of 0.05 and a log LDA score of 2.0. A total of 22 significant features was found and plotted in the form of a barplot. The differential CAZyme analysis showed 101 CAZymes to be differentially expressed in treatment and control. The top 20 are plotted in Figure 6. These differentially expressed families mainly included the GH family, which is mainly involved in the deconstruction of fibrous feed in the rumen. On the other hand, members of GT and CBM groups were also among the differentially expressed families. This indicated that the supplementation of additives modulated the expression of fibre-degrading enzymes in the rumen. The dominant phyla in the rumen of Holstein cattle include Bacteroidota, constituting about half, and Firmicutes, accounting for approximately a third of the total. Archaea are reported to constitute only a small quantity, equivalent to 0.5–1% of the total DNA in the rumen microbiome of Holstein cattle (25). Carbohydrate processing enzymes (CAZymes) are classified into families (CAZy families) based on their amino acid sequence similarity (26, 27). Archaea are notoriously known to encode few CAZymes, whereas bacterial species belonging to, e.g., *Bacteroidota* and *Firmicutes* are endowed with a strong palette of various CAZymes (Figure 7).

## Conclusion

The study explored the effects of feed additives on the rumen microbiome of buffaloes, highlighting significant changes in microbial community structure and functional capabilities. In the bacterial domain, *Bacteroidetes* and *Firmicutes* were the dominant phyla, with *Prevotella* as the most abundant genus, while other fibrolytic microbes displayed reduced abundance. These findings suggest the presence of alternate fiber-degrading pathways apart from traditional cellulolytic microbes such as *Fibrobacter* and *Ruminococcus*. Archaeal analysis revealed a reduction in methanogenic archaea, particularly *Methanobrevibacter*, in the treatment group, indicating the additive’s role in reducing methane production. Core microbiome analysis identified common genera but also highlighted novel unassigned microbes playing crucial roles in rumen function. KEGG pathway analysis showed consistent metabolic pathways across all groups, while CAZyme analysis revealed enhanced fiber-degrading enzyme expression in the treatment group. Overall, the study demonstrates that phyto feed additives modulate the rumen microbiome, reduce methane production, and potentially enhance fiber utilization, contributing to sustainable livestock management practices.

## Data Availability

The data presented in the study are deposited in NCBI BioSample repository, under the accession numbers SAMN52663503 and SAMN52663504.
